# Multi-Stage Feature Selection by Using Genetic Algorithms for Fault Diagnosis in Gearboxes Based on Vibration Signal

**DOI:** 10.3390/s150923903

**Published:** 2015-09-18

**Authors:** Mariela Cerrada, René Vinicio Sánchez, Diego Cabrera, Grover Zurita, Chuan Li

**Affiliations:** 1 Control Systems Department, Universidad de Los Andes, Mérida 5101, Venezuela; 2 Mechanical Engineering Department, Universidad Politécnica Salesiana, Cuenca 010150, Ecuador; E-Mails: rsanchezl@ups.edu.ec (R.V.S.); dcabrera@ups.edu.ec (D.C.); gzuritav@ups.edu.ec (G.Z.); 3 Chongqing Key Laboratory of Manufacturing Equipment Mechanism Design and Control, Chongqing Technology and Business University, Chongqing 400067, China; E-Mail: chuanli@21cn.com; 4 Mechanics Department, Universidad Nacional de Educación a Distancia, Madrid 28040, Spain

**Keywords:** fault diagnosis, gearbox, vibration signal, feature selection, genetic algorithms, neural networks

## Abstract

There are growing demands for condition-based monitoring of gearboxes, and techniques to improve the reliability, effectiveness and accuracy for fault diagnosis are considered valuable contributions. Feature selection is still an important aspect in machine learning-based diagnosis in order to reach good performance in the diagnosis system. The main aim of this research is to propose a multi-stage feature selection mechanism for selecting the best set of condition parameters on the time, frequency and time-frequency domains, which are extracted from vibration signals for fault diagnosis purposes in gearboxes. The selection is based on genetic algorithms, proposing in each stage a new subset of the best features regarding the classifier performance in a supervised environment. The selected features are augmented at each stage and used as input for a neural network classifier in the next step, while a new subset of feature candidates is treated by the selection process. As a result, the inherent exploration and exploitation of the genetic algorithms for finding the best solutions of the selection problem are locally focused. The approach is tested on a dataset from a real test bed with several fault classes under different running conditions of load and velocity. The model performance for diagnosis is over 98%.

## 1. Introduction

Industrial environments have constantly increasing requirements for the continuous working of transmission machines. This is why new proposals for building fault diagnostic systems with low complexity and adequate accuracy are highly valuable. Invaluable studies to detect gear faults by using standard diagnostics techniques based on signals have been widely developed [1]. In gear fault diagnosis, several analysis techniques have been used, such as wavelet transform [2,3,4], Kalman filtering analysis [5], particle filtering [6], blind source separation techniques [7] and generalized synchrosqueezing transform [8], among others techniques. The availability of an important number of condition parameters that are extracted from rotating machinery signals, such as vibration signals, has motivated the use of machine learning-based fault diagnosis, where common approaches use neural networks (NN) and related models, because of the simplicity for developing industrial applications [9,10,11,12,13,14,15,16]. These approaches have been very useful for implementing condition-based maintenance (CBM), as is presented in Jardine *et al.* [17].

Condition parameters are mostly related to time and frequency domains, but parameters from the time-frequency domain are also used in order to enhance the condition data to be processed by the diagnosis algorithms. Then, the high dimensionality of the input vector for machine learning-based diagnosis applications is a problem that should be addressed, because this high dimensionality can lead to over-fitted models. This is a well-known problem. On the other hand, the failure nature could be associated with certain condition parameters, and in the case of incipient failures, it is not clear what are the best condition parameters providing good diagnostic information.

Taking into account the availability of a large number of parameters as condition candidates for fault diagnosis, the problem of parameter selection is still an open research area in machine learning-based diagnosis. In most cases, the feature selection process has been treated as a dimensionality reduction problem by using principal component analysis, multidimensional scaling, factor analysis, projection pursuit and other linear and non-linear techniques [18]. However, the physical meaning of the extracted original condition parameters may be lost by creating new artificial features. Some effort to use dimensionality reduction techniques for finding the best subset of the original features has been performed in Bartkowiak and Zimroz [19], by using multivariate linear regression and variable shrinkage.

Feature selection aims to feature elimination to remove irrelevant features, by using wrapper, filtering or embedded methods [20]. Some heuristic search algorithms can be used for feature selection in a wrapper approach. The research in Saravanan *et al.* [12] and Saravanan *et al.* [13] uses a decision tree for developing a first approximate diagnostic model, and the features that are selected for the decision algorithm are stated as the best input features for the diagnosis model based on a neural network. On the other hand, genetic algorithms (GA) have been widely used in wrapper approaches for dimensionality reduction [21]. In that framework, for the diagnosis of the machinery condition, GA may be used in order to select the best features, aiming to improve the diagnosis model accuracy. Classically, GA are applied to the whole set of feature candidates, as is presented in Hajnayeb *et al.* [10], Samanta [22], Samanta [23] and Samanta *et al.* [24]. In such a case, the computational effort to execute the GA and also the effort to reach the optimal solution may be hard when a high-dimensional space is analyzed.

In order to improve the searching process, stage-based approaches have been proposed for feature selection. In Karabadji *et al.* [25], a wrapped approach is presented for selecting the best subset of features, aiming to improve the performance of the tree-based diagnosis model by using a research graph. As a result, each possible feature subset is a node of the graph, and the searching space exploration is performed by using an iterative algorithm that alternates the candidate generation and evaluation phases. In Rajeswari *et al.* [26], another stage-based selection is presented by using GA and a rough set-based approach; in this case, GA operates on the population of independent features that are generated by a filter approach based on a rough set by evaluating the dependency factor between features. Zhang *et al.* [27] presents multiple feature selection models in order to rank the feature candidates, and each set of candidates is re-ranked by using a weighted voting scheme based on the classifier performance. In the second stage, the number of re-ranked candidates is minimized by using two wrapper models. In Li *et al.* [28], two-stage feature selection has been proposed for fault diagnosis of a gearbox based on mutual information and GA. The feature candidates have been selected with mutual information in the first stage; this is a filtering method, and the optimal feature subset is obtained by using GA in the second stage, this being a wrapper method. In Yang *et al.* [29], an iterative wrapper approach is also developed; the main idea is the splitting of the available data into several subsets, and one classifier for each set of data is proposed. Thereafter, the obtained classifiers are validated on the unbalanced test dataset, and the classification distributions are normalized and combined. The area under the ROC curve is calculated as the fitness indices for feature selection, and an algorithm based on GA is used for feature selection. On the other hand, hybrid approaches, as presented in Yang *et al.* [30], have been also proposed to solve the problem of feature selection in the fault diagnosis of rotating machinery. In this work, the concepts of similarity, feature re-ranking and redundancy evaluation are properly used from multiple clustering solutions for feature selection.

The previous works show that, nowadays, GA is still a useful approach for feature selection, but some problems for finding good solutions could arise when a high-dimensionality searching space is treated. Two problems could be highlighted: (i) GA are highly dependent on the initial conditions; then, exploration of the solution candidates should be carefully performed; and (ii) the inherent random nature of the GA could lead to more exhaustive exploration on the solution space, and the convergence could be slow. In this sense, good results by using GA are based on a balance between exploration and exploitation.

This work aims to improve the exploration and exploitation during the searching process, in order to discover the subset of condition parameters providing useful diagnostic information. Exploration is posed through a multi-stage approach to enhance the capability for showing different subspaces of the whole available space. Exploitation is treated by extending the best solution in the previous stage with additional information related to other solution subspaces in the new stage. The entire set of extracted condition parameters from the time, frequency and time-frequency domains, called the features for the diagnosis problem, is split into different disjoint subsets. In each stage of the selection process, the GA is performed only on a subset, and the selected features extend the previous selected inputs to the classifier. As a result, the input features are augmented in each stage with the selected features, and these new augmented features are considering as fixed input to the next stage, where a new subset of feature candidates is processed by the GA. In this way, the main contribution of our approach aims to discover the most important features of each subset. This is significant, because the searching space is delimited in each stage, and as a consequence, the exploration and exploitation for searching the best solution is improved on a local feature subspace.

A classical neural network is used as a classification model; then, in addition, the optimization process at each stage is also applied for selecting the best number of hidden neurons. The approach is validated with data from a test bed that simulates real industrial environments for several fault occurrences under different running conditions of load and velocity. The classification performance is over 98% with 45% of the available features. This result is compared to the classical feature selection by using GA on the whole set of available condition parameters, where classification performance is over 97% with 53% of the available features. The proposed feature selection is a simple, but useful wrapper approach, according to the result analysis.

This paper is organized as follows. Section 2 presents methods and materials supporting the proposed approach. Conceptual foundations on GA and NN are briefly depicted; the details on the experimental procedure to collect the data and feature extraction process are shown. Finally, the multi-stage feature selection approach is developed. Section 3 discusses and compares the obtained results regarding the use of features from time and frequency domains and only features from time-frequency domains, with the classical one-stage GA algorithm. Additionally, some comparisons and analysis using feature selection through the importance variables from Random Forest algorithm are also provided in this section. Section 4 presents the conclusions of this work.

## 2. Experimental Section

### 2.1. Theoretical Background

This section presents the basic conceptual foundations on GA as a technique for multi-objective optimization and NN as a classifier for fault diagnosis. More detailed foundations about these techniques can be found in the references.

#### 2.1.1. Genetic Algorithms

**Figure 1:**
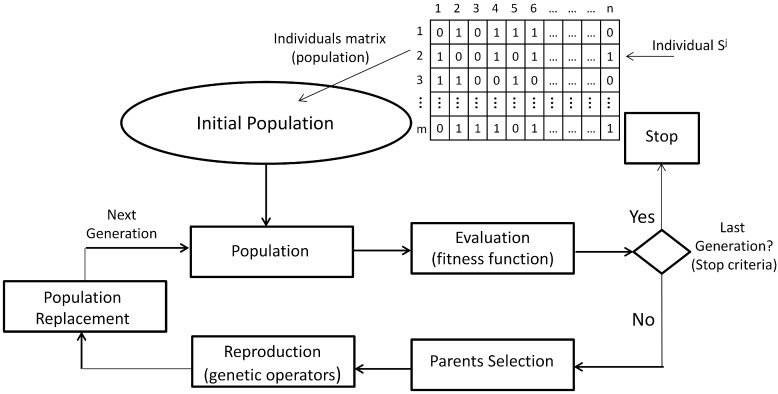
General cycle of the GA.

The GA are probabilistic search algorithms that emulate the biological population evolution of individuals, by applying operators called genetic operators, allowing recombination of such individuals in order to strengthen them regarding an indicator of individual quality. Based on biological theories of genetic inheritance and survival of the best individuals, the GA have been popularly used for solving NP-complete optimization problems, and they constitute the central paradigm of evolutionary computing (EC). The basic algorithm that determines the operation of EC algorithms, including the GA, is shown in Figure 1. It contains the following key elements [31].

(1) Individual encoding: In the GA technique, each individual is associated with a structure and a content. The structure refers to the format, and the content refers to the information provided by the individual in each component of its structure. In general, an individual $S^{j}$ is encoded as a binary string of length *n*, where each binary element is called a gene. However, individual encoding can be adapted to the nature of the solutions for the particular problem.
(2) Initial population: This is composed of individuals of the first generation of possible parents. In this way, the initial population *P* is the set $P=\left\{S^{j}\right\}$, j=1,...,m, which can be seen as a binary matrix, where each row is one individual and each column is the value of the corresponding gene.
(3) Fitness function: The value of the fitness function *f*, or the evaluation function, is a measure of each individual performance in the GA. In general, the fitness function *f* is a mapping f:P→ℜ, which assigns to each individual a real value according to its performance for solving the optimization problem. A fitness scaling is performed sometimes to avoid the dispersion of the function values.
(4) Parent selection: The process for selecting the best individuals for the next generation should be guided, based on the values of the fitness function. There are different selection mechanisms, most of them based on the relative probability of the selection $p_{j}$ of an individual $S^{j}$.
(5) Genetic operators and reproduction: The reproduction is achieved by applying genetic operators to produce new individuals with improved genetic material. Usually, the crossover and mutation operators are applied; however, other operators also exist, and they can be more appropriate in specific problems.
(6) Population replacement: Replacement strategies should aim to maintain diversity in the population, as well as to improve the evaluation of the fitness function of the individuals of the new population. Once new individuals have been generated, they replace only a part of the parents for the new generation. Other replacement strategies are the direct replacement, where the children replace the parents in the next generation, and the elitist replacement, where a fraction of the best individuals goes directly to the next generation.

GA runs in an iterative way until reaching the best solution according to some stop criteria, and each iteration executes the cycle in Figure 1. The specification of the GA in our approach is detailed in Section 2.3.

#### 2.1.2. Artificial Neural Networks

**Figure 2:**
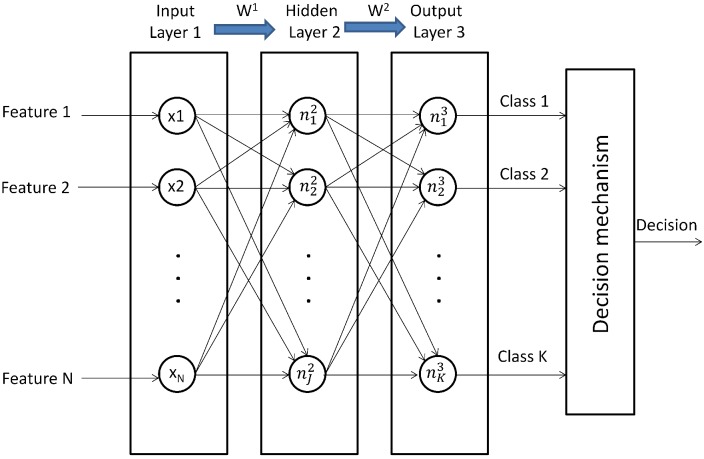
Basic architecture of an NN for classification.

Artificial neural networks or neural networks (NN) are computational information models emulating biological neural networks by defining non-linear relationships between a set of data called “the inputs” and “the outputs”. Then, the information processing is given through non-linear functions, and a learning process is used for adjusting the functions parameters in order to achieve a good explanation between inputs and outputs. Figure 2 shows the basic architecture of an NN for classification purposes; it is composed of three layers with neurons: the input layer that is associated with the input features, the hidden layer with neurons that are associated with $n_{j}^{1}$ functions j=1...,J and the output layer with neurons that are associated with $n_{k}^{2}$ functions k=1...,K, each neuron in the output layer proposing the membership to the class $C_{k}$ for the input features. There are weighted linkages between neurons in two successive layers; then, $W^{l}$ is the vector of weights controlling the function mapping from layer *l* to layer l+1. These vectors are properly arranged into the matrix *W*. In general, for a layer *l* and neuron *j*:

(1) $$n_{j}^{l}=f\left(w_{j0}^{\left(l-1\right)}x_{0}^{\left(l-1\right)}+w_{j1}^{\left(l-1\right)}x_{1}^{\left(l-1\right)}+...+w_{jN}^{\left(l-1\right)}x_{N}^{\left(l-1\right)}\right)$$

Where the vector $x^{\left(l-1\right)}=\left(x_{0}^{\left(l-1\right)},x_{1}^{\left(l-1\right)},...,x_{N}^{\left(l-1\right)}\right)$ is the output of the previous layer, Nis the number of neurons in the previous layer, $x_{0}^{\left(l-1\right)}$ is the bias, $w_{ji}^{\left(l-1\right)}$ is a weight, w∈ℜ, and f(•) is called the activation function. Usually, the unipolar sigmoid function is commonly used as the activation function for each neuron $n_{j}^{l}$.

Consider the data with *m* samples of a couple $(x^{i},y^{i}),i=1,...,m$; $x^{i}$ is a vector of *N* features, *i.e.*, $x^{i}=\left(x_{1}^{i},x_{2}^{i},...,x_{n}^{i}\right)$, and $y^{i}=\left(y_{1}^{i},y_{2}^{i},...,y_{K}^{i}\right)$ is the output vector where $y_{k}^{i}=1$ indicates that the vector of features $x^{i}$ is associated with the class $C_{k}$, and $y_{k}^{i}=0$ otherwise. Most of the problems in data-driven multi-class classification, as fault diagnosis, may be solved with a classical feedforward NN by using the backpropagation algorithm with the gradient descent method for adjusting the vector of weights $W^{l}$ in order to minimize the cost function *J* in Equation (2) [32,33]:

(2) $$J\left(W\right)=-\frac{1}{m}\left[\sum_{i=1}^{m}\sum_{k=1}^{K}y_{k}^{i}\;log\left(h_{W}{\left(x^{i}\right)}_{k}\right)+\left(1-y_{k}^{i}\right)\;log\left(1-h_{W}{\left(x^{i}\right)}_{k}\right)\right]$$

where $h_{W}{\left(x^{i}\right)}_{k}$ is the output function of the neuron *k* in the output layer.

Given the dataset with *m* samples $\left(x^{i},y^{i}\right)$, the algorithm for training an NN for classification purposes is widely known, and it is summarized as follows, [34]:

(1) Randomly initialize the weights of each layer $W^{l}$, l=1,...L, where $w_{ji}$ is the weight from the neuron $n_{i}^{\left(l-1\right)}$ to the neuron $n_{j}^{l}$.
(2) Compute the feedforward propagation to obtain $h_{W}{\left(x^{i}\right)}_{k}$.
(3) Compute the cost function J(W).
(4) Run the backpropagation algorithm to compute $\frac{\partial }{\partial w_{ji}^{l}}J\left(W\right)$.
(5) Use the gradient descent method for adjusting the weights $w_{ij}^{l}$ according to the equation $w_{ij}^{l}:=w_{ji}^{l}-\alpha \frac{\partial }{\partial w_{ji}^{l}}J\left(W\right)$.

In our approach, NN is used as the diagnoser, and the precision of the diagnostic result that is associated with each individual is used as the input for processing the fitness function in GA. The details about coupling NN to the optimization problem of feature selection by using GA is presented in Section 2.3.

### 2.2. Measurement Procedure and Feature Extraction

**Figure 3:**
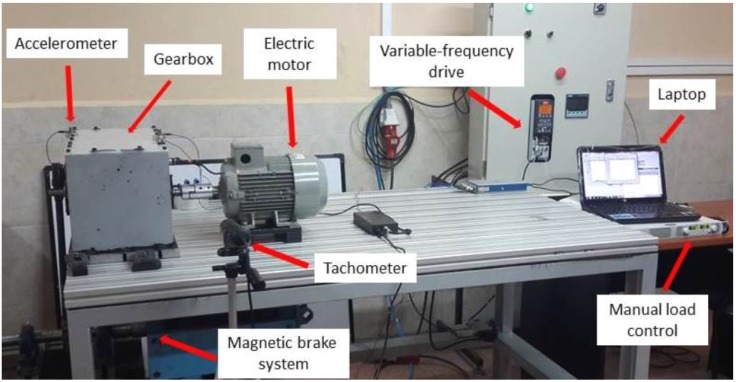
Vibration analysis laboratory at the Universidad Politécnica Salesiana in Cuenca, Ecuador.

This section presents the experimental setup to build the data matrix that will be used in our approach. Our test bed in Figure 3 has been designed to simulate real faults that can occur in industrial environments. The rotation motion of the equipment is generated by a 1.1-kW motor powered by three-phase 220 V at 60 Hz with a nominal speed of 1650 rpm. The torque motion is transmitted into a gearbox, where several gears fault configurations are assembled. At the end of the gearbox shaft, the torque is transmitted to a pulley, which is part of the magnetic brake system. The magnetic brake function is to control different loads according to the measurement settings. A variable-frequency drive was used to generate different speeds. Table 1 and Table 2 show the details of the different gear faults under study.

The vibration analyzer and digital balancer Digivive MX-300 was used to collect the raw signals; the data acquisition software was performed by Digivibe MX 5.14. In order to record the spur gear vibration signal, the accelerometer with a sensitivity of 330 mV/g was vertically allocated. The measurements were conducted at different speeds (300, 600, 900, 1200 and 1500 rpm) and with different break loads of about 10%, 50% and 90% regarding the maximum power of the motor. The total number of measured signals, with different measurements settings, was up to 1200 signals (8 gear faults, 3 loads, 5 speeds, 10 measurements repetitions for each case on 2 s, with 1-s interval between samples). The sampling frequency was about 11,025 Hz.

**Table 1:** Simulated gear faults.

Label	Description
f0	Normal
f1	Gear crack 0.5 mm
f2	Gear tooth breakage 10%
f3	Pinion pitting
f4	Pinion with face wear 0.5 mm
f5	Gear misalignment
f6	Gear tooth breakage 50%
f7	Gear tooth breakage 100%

**Figure 4:**
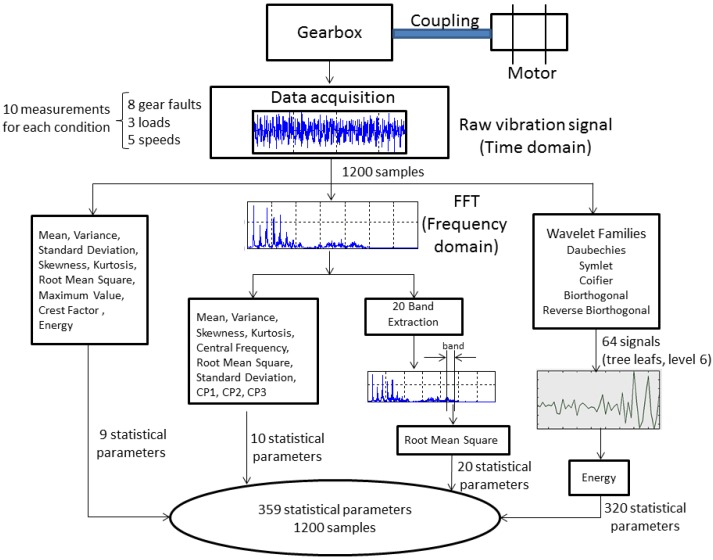
Feature extraction process from the vibration signal.

The signal processing for extracting the most common condition parameters was performed in MATLAB${}^{©}$. Each raw vibration signal was treated to compute condition parameters from the time, frequency and time-frequency domains. In particular, wavelet packet decomposition (WPD) was used to extract condition parameters from each signal at the last level of the decomposition. Several works in the literature propose combining a large number of condition parameters as feature candidates, because different faults could have different effects on certain condition parameters. In the case of incipient failures, it is not clear what are the best condition parameters providing good diagnostic information. The next sections describe the feature extraction procedure for each domain, and it is summarized in Figure 4, for some vibration signals. At the end of the process, we have a data matrix with 1200 rows and 359 columns; each row is a sample, and each column is the value of the corresponding condition parameter.

**Table 2:** Gear faults.

Image	Description
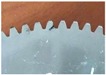	Gear crack 0.5 mm
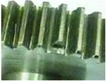	Gear tooth breakage 10%
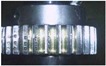	Pinion pitting
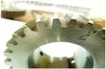	Pinion with face wear 0.5 mm
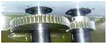	Gear misalignment
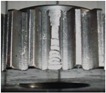	Gear tooth breakage 50%
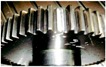	Gear tooth breakage 100%

#### 2.2.1. Condition Parameters on the Time and Frequency Domains

Nine classical condition parameters were obtained by statistical analysis from the time domain, such as: mean, variance, standard deviation, skewness, kurtosis, root mean square (RMS), maximum value, crest factor and energy. In the case of the frequency domain, the fast Fourier transform (FFT) is applied to the time signal, and ten condition parameters are calculated, such as: mean, variance, skewness, kurtosis, central frequency, root mean square (RMS) and the standard deviation. Equations for computing these parameters can be found in [35,36]. Additionally, another three condition parameters in Equations (3)–(5) have been included, denoted as $CP_{1}$, $CP_{2}$ and $CP_{3}$ [35]:

(3) $$CP1=\frac{\sum_{k=1}^{K}{\left(f_{k}-FC\right)}^{3}s\left(k\right)}{K{\left(STDF\right)}^{3}}$$

(4) $$CP2=\frac{STDF}{FC}$$

(5) $$CP3=\frac{\sum_{k=1}^{K}{\left(f_{k}-FC\right)}^{\frac{1}{2}}s\left(k\right)}{K\sqrt{STDF}}$$

where s(k) is the spectrum for k=1,...,K, *K* is the number of the spectrum lines, $f_{k}$ is the frequency value of the k-th spectrum line, STDF is the standard deviation for frequency and FC is the frequency center.

Another condition parameter on the frequency domain is the RMS value on a specific frequency band. The rationale for using frequency bands is because a fault can generate clear changes in the vibration amplitude in a band where, usually, this amplitude is non significant in the case of no faults. In this work, the RMS value on twenty frequency bands on the whole frequency range of 3660 Hz was calculated, each band a size of 183 Hz; however, different sizes can be analyzed to identify more specific bands providing diagnostic information.

#### 2.2.2. Condition Parameters on the Time-Frequency Domain

**Figure 5:**
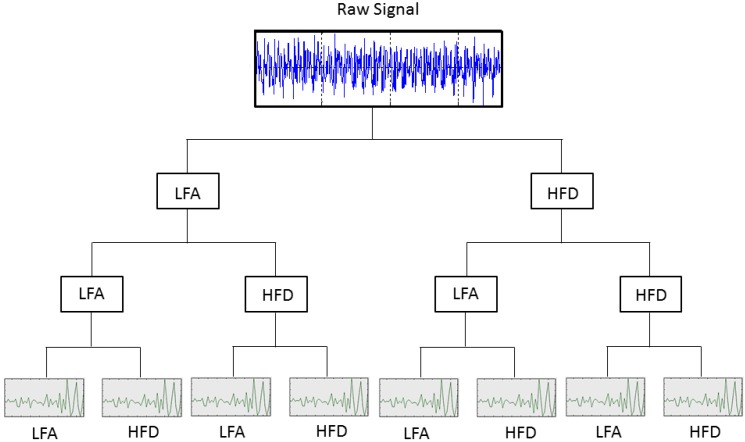
The wavelet packet decomposition.

The time raw signals were used as input data for the wavelets analysis, in order to obtain the energy of each wavelet coefficient, which are used as features in the diagnosis problem. Wavelet transform (WT) is a powerful tool that has attracted great attention in several fields, such as engineering, and, particularly, as a powerful analysis instrument for gear fault detection and diagnosis [3,4,37,38,39]. The analysis presented in Yan *et al.* [38] provides an extensive overview of some of the latest efforts in the development and applications of WT for fault diagnosis in rotating machinery. In the WPD framework, compression and de-noising ideas are exactly the same as those developed in the WT framework. The only difference is that WPD offers flexible analysis, because the details, as well as the approximations of the analyzed signal are split into tree-based decomposition. Figure 5 shows the decomposition of some raw signals until Level 3; the signals at the last level are the wavelet coefficients.

The wavelet function is designed to keep a balance between the time domain and the frequency domain. From the point of view of diagnosis, wavelet coefficients can have important diagnostic information to be exploited, and it could be different from the information in the RMS values of the frequency bands, because the information on the time domain is completely lost by the FFT analysis. More details about the use of the WT and WPD for fault diagnosis in rotating machinery are in [38,40].

Five mother wavelets are used for extracting condition parameters for our approach: Daubechies (db7), symlet (sym), Coifier(coif4), biorthogonal (bior6.8) and reverse biorthogonal (rbior6.8). In a wavelet analysis, different combinations of bases could produce a more desirable representation for a particular signal, as different mother wavelets applied to the same signal could produce different results. There are no standard or general methods to select mother wavelets. The intention to use several wavelets is to find out the most suitable wavelet for our application. The coefficients are collected from Level 6 for each mother wavelet; then, $2^{6}$ coefficients are obtained. The energy has been calculated for each coefficient, and they complete the feature vector for diagnosis.

### 2.3. Multi-Stage Feature Selection Based on GA with NN Classifiers

**Figure 6:**
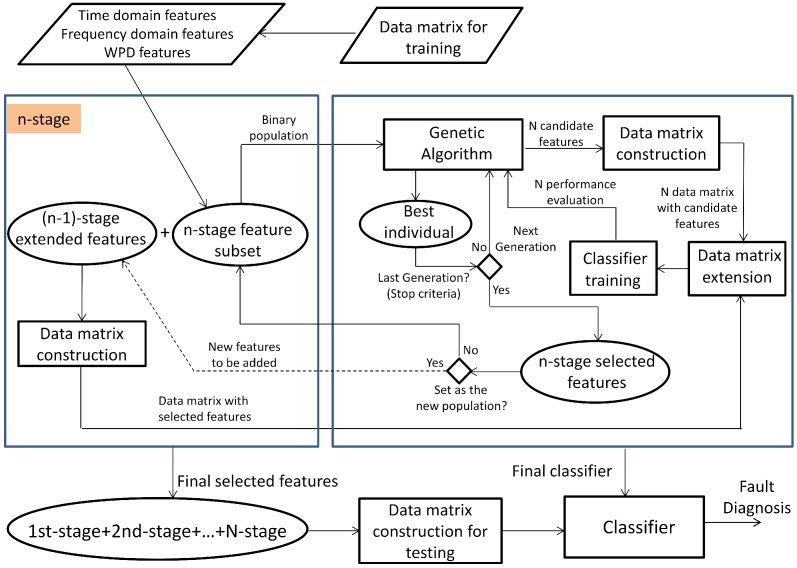
Multi-stage approach for feature selection in fault diagnosis.

This section presents the multi-stage feature selection approach and the details about the GA design. Figure 6 shows the architecture for implementing our approach. After developing the feature extraction in Section 2.2, a data matrix with 1200 samples (150 samples for each fault mode) and 359 condition parameters from the time, frequency and time-frequency domains are the input for the algorithm.

Firstly, the entire set PS of the available condition parameters cp is defined, $PS=\{cp_{1},...,cp_{i},...,cp_{n}\}$. This set is split into different disjoints subsets $PS_{k}=\left\{cp_{j}^{k}\right\}$, that is $PS=PS_{1}\dot{\cup }PS_{k}\dot{\cup }...\dot{\cup }PS_{l}$, k=1,...,l, where $\left|PS_{k}\right|=N$ and $\sum_{k=1}^{l}\left|PS_{k}\right|=n$. At each stage, one subset is selected to be processed by the GA according to the classical cycle in Figure 1. The details of each element of the algorithm are given in Section 2.3.1. The population is proposed from the available condition parameters in the selected subset $PS_{k}$, and the individuals matrix is treated. A data matrix with samples and the corresponding condition parameters are created for each individual to train an NN-based classifier. The performance for each individual is computed from the diagnostic precision of its corresponding NN, by using a validation dataset. Then, for each stage, the training phase for all NNs is performed in the classical cycle of GA until reaching the stop criteria. Once the best individual is selected, the subset of selected condition parameters is defined, and they can be considered as a partial fixed input to the classifier; or, also, they can be considered to propose the new population in the next step, according to the decision of the expert. For the next stage, the selected condition parameters extend the current partial fixed input in the previous step, and another subset $PS_{k}$ is processed. All of the subsets $PS_{k}$ that have been processed in previous stages are not taken into account in the next ones.

The selection process in Figure 6 runs in an iterative way until processing all of the subsets $PS_{k}$. At the end of this process, the set of the selected features is obtained, and the diagnosis model based on NN is reached.

#### 2.3.1. GA Design

**Figure 7:**
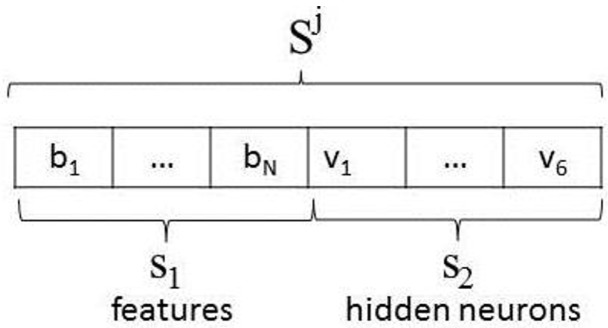
Individual encoding.

The GA was designed as follows:

(1) Individual encoding: Two partial solutions are encoded in this approach: the features to be selected and the number of hidden neurons in the NN. Figure 7 shows the encoding for only one hidden layer. In this way, every possible solution $S^{j}$ is a vector with the first chain of bits $s_{1}$ encoding the condition parameters in the subset $PS_{k}$, where the bit $b_{j}$, j=1,...,N, can be $b_{j}=1$, which indicates the selection of the condition parameter $cp_{j}^{k}$, or $b_{j}=0$ otherwise. The second chain of bits $s_{2}$ is composed of six bits $v_{j}$, 0 or 1, encoding the binary number to propose 63 hidden neurons at most.

(2) Initial population: At each stage, a population P from the set $PS_{k}$ is generated with random selection of 0 or 1 for each bit $b_{j}$ and $v_{j}$.
(3) Fitness function: The performance of the classifier in the training and test phases is based on the mean square error value that is defined in Beleitesa *et al.* [41].
(4) Parent selection: Firstly, fitness scaling based on rank was used for defining the raw fitness value over a suitable range [42]. According to this scaling, an individual with a lower value of raw fitness is assigned to a higher value of scaling fitness; consequently, the individual has a higher probability to be chosen as a parent by the selection function. The rank is a function rank:f→N, which sorts individuals from 1 to *N* based on the best fitness value (lower value of the fitness function). Once the individuals are ranked, their new fitness $f_{ranked}$ is recalculated according to Equation (6):
  (6) $$f_{ranked}=p-2\frac{\left(r-1)(p-1\right)}{P_{s}-1}$$
  where *r* is the rank of the individual, *p* is the desired selective pressure and $P_{s}$ is the population size. The selective pressure is the probability that the best individual is selected regarding the average selection probability of the remaining individuals. The rank-based fitness scaling allows a selective pressure between about [1.0,2.0]. The uniform stochastic selection method was applied in this approach:
  (a) Determine the cumulative probabilities $q_{i}$ based on the value $f_{ranked}$ of each individual, as follows:

					
                      $q_{0}\leftarrow 0$
                    
                    
                      fori=1,...,N,
                    
                                $q_{i}\leftarrow q_{i-1}+f_{ranked}^{S^{i}}/\sum_{j=1}^{N}f_{ranked}^{S^{j}}$
                    
                    
                      end
  (b) Select *K* parents, in the following manner:

				  
                      Fori=1,...,K
                    
                              $r\leftarrow random(0,q_{N})$
                    
                                
                            $parent^{i}\leftarrow S^{i}\;\mathrm{if}\;q_{i-1}<r<q_{i}$
                    
                    
                      end
(5) Genetics operators: Crossover and mutation operators were applied. The crossover fraction was set at 80%, that is the number of children that will be obtained from crossover. The crossover point selection was performed by applying a random scattered selection, as follows:
  (a) Select Father 1
  (b) Select Father 2
  (c) Generate a random binary vector *v* of N bits, that is $v_{i}=0$ or $v_{i}=1$
  (d) If $v_{i}=1$,
    - Gene *i* of Parent 1 is preserved,
      otherwise,
    - Replace the gene *i* of Parent 1 with the gene *i* of Parent 2.
    The 20% of the remaining population will be obtained by mutation, with a mutation rate of 0.05 for each gene of the selected father.
(6) Parent replacement: A direct replacement mechanism was used; 10% of the current population was selected by elitism, and they will be part of the next generation. The remaining individuals are replaced according to the fraction of children obtained by crossover and mutation.
(7) Stop criteria: The maximum number of generations, the cycles of GA in each stage, was selected as the stop criteria.

## 3. Results and Discussion

The proposed multi-stage selection approach was performed on the data matrix in Section 2.2. Each column of this matrix is the value of its corresponding condition parameter on the time, frequency and time-frequency domains. The 70% of the available samples was taken as the training set; 15% was assigned to each set for validation and testing. The validation set is used during the training phase for the NN, and the test set will be used for testing the performance of the final classifier at each stage, as presented in Section 3. A classical three-layer NN was used, as is illustrated in Figure 2.

At first, available conditions parameters cp define the set PS, with n=359. Two disjoint sets $PS_{1}$ and $PS_{2}$ are proposed, each one with condition parameters $cp_{j}^{k}$ from the time and frequency features for k=1 and the time-frequency features for k=2.

The multi-stage selection approach ran in three stages as follows:

(1) First stage: The subset $PS_{1}$ was processed at first, $\left|PS_{1}\right|=39$ (see Table 3). The initial population was 20 individuals; then, a binary matrix with 20 rows and 45 columns is defined, and 20 data matrices for training and validation are created for each individual. After the selection process by using GA, 18 features are selected with 43 hidden neurons. These features are fixed inputs for the NN-based classifier in the second stage.
(2) Second stage: The subset $PS_{2}$ was processed at this stage, $\left|PS_{2}\right|=320$ (see Table 3). At this stage, the selection process is addressed for detecting the relevance of the condition parameters from each wavelet family. In this sense, the partial solution related to the input features is encoded as a binary sequence of five bits, where $b_{i}=1$ indicates that all of the energy parameters from wavelet family coefficients are selected as inputs, and $b_{i}=0$ indicates that all parameters are excluded. The initial population was 20 individuals; then, a binary matrix with 20 rows and 11 columns is defined, and the corresponding data matrices for training and validation are created for each individual. The selection process only runs on the partial inputs to the NN that are being optimized. Finally, all parameters from wavelet family rbior6.8 are discarded, and 30 hidden neurons are selected. At this stage, according to the decision of the user, the selected condition parameters are not taken for extending the previous fixed inputs (18 features in the first stage), and a new subset $PS_{3}$ containing parameters from the remaining wavelet families is created. This subset will be treated in the third stage.
(3) Third stage: The subset $PS_{3}$ was processed at this stage, $\left|PS_{3}\right|=256$ (see Table 3). The initial population was 100 individuals; then, a binary matrix with 100 rows and 262 columns is defined, and the corresponding data matrices for training and validation are created for each individual. The selection process runs on the partial inputs to the NN. As a result of the selection process, 144 parameters are selected and accepted to extend the previous 18 fixed inputs. The proposed number of hidden neurons was 36. All subsets $PS_{k}$ have been processed, and the multi-stage process is completed.

**Figure 8:**
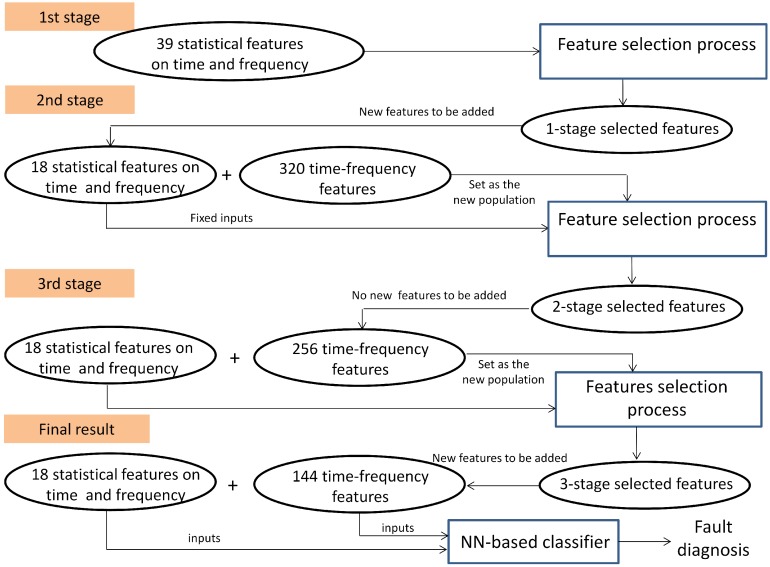
Case study: Final results.

**Figure 9:**
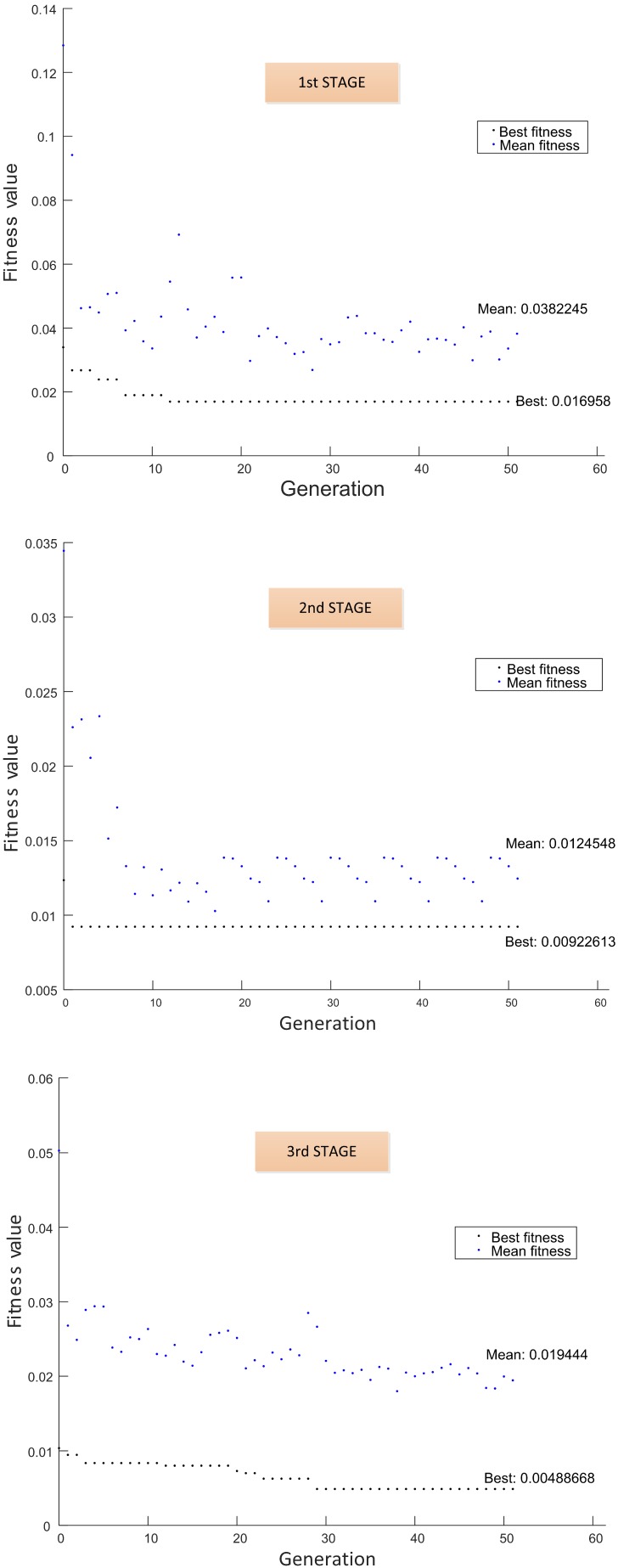
Fitness function performance.

The final set of classifier inputs, that is the features for the diagnostic problem, is composed of 162 features with 36 hidden neurons. This multi-stage process is illustrated in Figure 8 and Figure 9, showing the fitness function evolution. The fitness value was improved from 0.016958 in the first stage to 0.00488668 in the third stage. The best set of the selected features is summarized in Table 4.

**Table 3:** Sets of the initial population. db7, Daubechies; sym3, symlet; coif4, Coifier; bior6.8, biorthogonal; rbior6.8, reverse biorthogonal.

Number of Stages	Number of Features	Condition Parameters
1	39	20 RMS values from frequency bands + 10 from the frequency domain
		+ 9 from the time-domain
2	320	energy from wavelets coefficients (db7 + sym3 + coif4 + bior6.8 + rbior6.8)
3	256	energy from wavelets coefficients (db7 + sym3 + coif4 + bior6.8)

**Table 4:** Best individual by using multi-stage selection.

Parameters	Total Number	Description
RMS from frequency bands	12	−183 Hz (1st band)
		733 Hz to 915 Hz (5th band)
		915 Hz to 1098 Hz (6th band)
		1281 Hz to 2702 Hz (8th band to 14th band)
		2745 Hz to 3111 Hz (16th band to 17th band)
Frequency	2	Standard deviation
		Skewness
Time	4	Mean
		Standard deviation
		Root Mean Square
		Crest Factor
Energy from Wavelets	144	36 coefficients from db7
coefficients		37 coefficients from sym3
		35 coefficients from coif4
		36 coefficients from bior6.8

**Figure 10:**
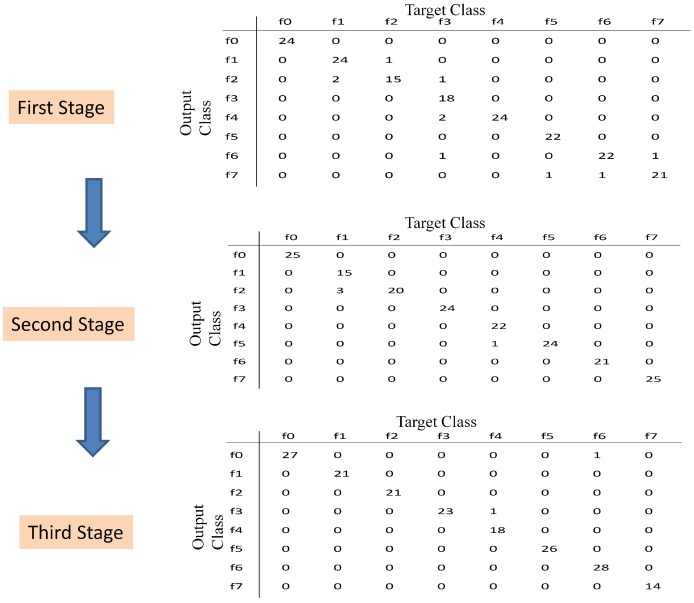
Confusion matrix for the test set, at each stage.

This section presents the results after applying the proposed multi-stage feature selection. The proposed classifier at each stage is evaluated on the data in the test set. Figure 10 illustrates the sequence of the obtained confusion matrix; there are ten misclassified samples in the first stage, four misclassified samples in the second stage and only two misclassified samples in the third stage. The classical $F_{1}$-score is used as metric to measure the performance of the classifiers. Table 5 shows the final results where the $F_{1}$-score is improved from 0.9444 to 0.9889. This result shows that by aggregating new selected features and by adjusting the number of hidden neurons at each stage, the performance of the NN-based classifier is improved. Moreover, the use of only the time and frequency condition parameters is not enough to obtain a good performance in classification, as highlighted by the result in the first stage.

The previous results are compared to the classical GA-based feature selection on the entire set of available condition parameters, also considering the tuning of the hidden neurons’ number. The GA was applied on the set PS; 193 condition parameters were selected with 50 hidden neurons and $F_{1}=0.9778$. The reported result in Stage 3, by previously eliminating the parameters from the rbior6.8 wavelet family (see Table 5), is better than the obtained one from the entire set PS.

**Table 5:** Classifier performance by using multi-stage selection.

Number of Stages	Number of Fixed Features	Number of Available Initial Features	Number of Final Features	Number of Hidden Neurons	$F_{1}$-Score
1	0	39	18	43	0.9444
2	18	320	274	30	0.9778
3	18	256	162	36	0.9889

One additional experiment was developed to show the rationale of the individuals encoding in the second stage of our approach. One new initial set with 320 features from time-frequency data (by using the wavelets families db7, sym3, coif4, bior6.8 and rbior6.8) was treated by the classical GA, including the tuning of the hidden neurons’ number. As a result, 156 condition parameters were selected with 63 hidden neurons, and $F_{1}=0.9611$. This result proposes a large number of hidden neurons regarding the proposed ones in the second stage, and the performance is not better than the result in the second stage, even considering condition parameters from the wavelet family rbior6.8. Table 6 summarizes the experiments by using classical GA-based feature selection, and by comparing to Table 5, the proposed multi-stage approach using GA is more useful than the classical approach.

**Table 6:** Classifier performance by using classical feature selection with GA.

Number of Initial Features	Number of Final Features	Number of Hidden Neurons	Precision	Sensibility	$F_{1}$-Score
320	156	63	0.9611	0.9611	0.9611
359	193	50	0.9778	0.9778	0.9778

The results obtained with the GA-based multi-stage approach for feature selection was compared to the selection through the random forest (RF) algorithm. RF is an algorithm based on decision trees that uses a bagging strategy for improving the variance by decreasing the correlation between the trees. In RF, *k* decision trees are built and trained with bootstrap sample versions of the original training data. Then, given a new input, the estimated class is obtained from a voting process that is executed over the prediction given by each tree [43]. This algorithm is also suitable for obtaining the most representative attributes, since each decision tree computes the contributed information by each attribute to the classes. Therefore, in RF, the information of each attribute over all of the trees can be averaged in order to rank the important variables. The information degree based on entropy is widely used for ranking the variables. If the information degree is high, the attribute is more significant; then, RF is used as the feature selector [44].

**Figure 11:**
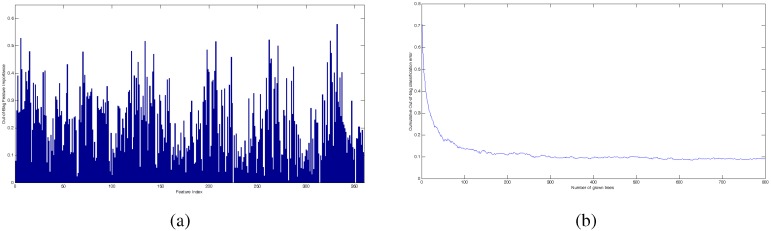
(**a**) Importance variables computed by the RF algorithm; (**b**) out-of-bag error in the training phase.

Figure 11a shows the importance variables for our case study, over the entire set of 359 features. The RF was run with 800 trees, and the performance for the cumulative out-of-bag error during the training is presented in Figure 11b. Out-of-bag error is a measure of the classification performance calculated with the samples that are out of the bootstrap samples used for growing each tree. In order to compare to the results in Table 5 and Table 6, we have selected the same number of features that have been selected by the multi-stage approach (162 features; see Table 5) and the best selection with the classical approach by applying GA over the entire set of 359 attributes (193 features; see Table 6). Additionally, we have also analyzed 6, 31 and 88 features with the information degree greaterthan 0.5, 0.4 and 0.3, respectively (see Figure 11a). On the other hand, because the RF was applied over the entire set of features, we have taken the number of neurons in Table 5 (50 hidden neurons) as a reference in the next experiments. Then, we have run two experiments with 40 and 60 hidden neurons, respectively.

Table 7 shows the precision in classification for the test set, with the selected features by RF, including the entire set of 359 features. At first result, NN needs more than 40 hidden neurons to improve the classification precision with the features selected by RF. Furthermore, the precision obtained with the best 162 features with our approach is better than the results with 162 features in Table 7. The same result is observed with 193 features. In general, for this case study, the results with the multi-stage approach using GA, even with the classical approach, are better than the results with RF-based feature selection. The selection of the best features and the best number of hidden neurons in NN applications is a classical problem that is analyzed simultaneously. The best selection of both components leads to the good performance of NN-based classifiers. The features that have been selected by the RF algorithm could need more analysis for setting the best number of hidden neurons, in the context of using NN.

**Table 7:** Classification performance by using RF-based feature selection and the NN-based diagnoser.

Number of Features	Precision with 40 Hidden Nodes	Precision with 60 Hidden Nodes
6	0.5000	0.5185
31	0.8278	0.8284
88	0.9037	0.9111
162	0.9389	0.9593
193	0.9481	0.9585
359	0.9481	0.9593

Table 8 presents the selected features with RF and the corresponding number of coincident features by using the multi-stage approach with GA. We can see that RF has selected 23 coefficients from the mother wavelets “rbior6.8” and no features from the time domain. This result is quite different from the results with our multi-stage approach, which has discarded the coefficients from “rbior6.8” and selected four condition parameters of the time domain. RF has selected the same number of 12 frequency bands, but only eight coincident coefficients. In the frequency domain, only the standard deviation has been the selected feature. Finally, 22 coefficients have been selected by the two methods from each coincident mother wavelet.

Previous results show that both methods produce different selections, and they are compared only based on their performance. Some aspects can be discussed in order to establish some analogies and differences between them. Feature selection with RF is an attractive method, because it is based on a metric that is easily calculated with low computational effort, regarding the approaches based on GA. The metric value is estimated from the out-of-bag samples, and the goodness of this value depends on the exhaustive search to find the best disjoint regions associated with a class, over a subset of features selected at random for each tree. In this sense, the RF method performs a random process for selecting features, like the process performed by the GA in a classical manner. Our multi-stage approach differs from the procedure in RF, because it aims to provided a random search over a refined subset of features, by applying genetic operators that are more sophisticated random operations than those ones applied in RF.

**Table 8:** Best 162 features selected by RF.

Parameters	Total Number	Description	Number of Coincident Parameters
RMS from frequency bands	12	2nd band	0
		5th band to 7th band	2
		9th band	1
		11th band to 17th band	5
Frequency	1	Standard deviation	1
Time	0	——–	0
Energy from Wavelets	149	39 coefficients from db7	22
coefficients		39 coefficients from sym3	22
		24 coefficients from coif4	22
		24 coefficients from bior6.8	22
		23 coefficients from rbior6.8	0

## 4. Conclusions

In this work, a multi-stage feature selection approach by using GA has been proposed for designing fault diagnosis models. The approach aims to select, in each stage, the best features from a subset of candidate features that improve classification metrics on the diagnostic model. The selected features are considered as a partial input in the next stage, while a new subset is treated by the GA. As a result, the input features to the classifier are augmented at each stage with a partial solution of the optimization problem. With this approach, we aim to improve the exploration in the search of the optimal solution by analyzing local subspaces of the entire available space. Moreover, exploitation is also improved by extending the best solution in the previous stage with additional information in the new stage.

The case study is the feature selection for fault diagnosis of a spur gearbox, where there is a large number of condition parameters as feature candidates. The selection of the parameters containing adequate diagnostic information is a very sensitive problem for implementing diagnosers with low complexity and high precision in industrial supervision applications.

The experimental results for this case study show interesting elements for fault diagnosis: (i) the use of the multi-stage approach improves the $F_{1}$-score of the diagnostic model regarding the classical approach considering the entire set of available features; in this case, the multi-stage approach proposes 162 features and 36 hidden neurons with an $F_{1}$-score over 98.9%; the classical approach proposes 193 features and 50 neurons with an $F_{1}$-score over 97.8%; (ii) the use of only the time and frequency condition parameters is not enough for obtaining a good performance in classification; and (iii) energy from wavelets coefficients, as condition parameters, improves the classification performance regarding the use of the time and frequency condition parameters. Comparison to the results by using RF-based feature selection shows that GA is still a good tool for optimizing search processes, and it can be implemented under new approaches to improve its performance.

Our framework is a general approach that can be applied to several case studies when a database with an adequate number of samples for NN-based classifiers is available. From the point of view of fault selection, in this case study, it was found that the feature candidates from the rbior6.8 wavelet family do not improve the useful diagnostic information. These results can lead to a study of the physical meaning of relevant condition parameters for fault diagnosis in spur gears.
